# Parturition and the Principles and Practice of Obstetrics

**Published:** 1849-12

**Authors:** 


*Parturition and the Principles and Practice of Obstetrics.*—By W. Tyler Smith, M. D., London. Lecturer on Obstetrics in the Hunterian school of Medicine. Philadelphia; Lea & Blanchard, 1849. 12mo. pp. 395.

This new work has been for some time on our table, and we have not, as yet, found time to give it the examination which it claims. The author treats of the various subjects embraced in parturition, in their relations to the functions of the reflex nervous system, as the latter are regarded by Marshall Hall. It is, in fact, a treatise on "reflex obstetrics," to quote the expression of the author, ând, the views set forth, as he states in his preface, are the results of "seven years' close and earnest attention to the subject." This being the case, it is presumable that it will, to say the least, repay the trouble of perusal.

